# Fungal Pathogens of Cacao in Puerto Rico

**DOI:** 10.3390/plants12223855

**Published:** 2023-11-15

**Authors:** Alina Sandra Puig

**Affiliations:** Foreign Disease-Weed Science Research Unit, United States Department of Agriculture-Agricultural Research Service, Fort Detrick, MD 21702, USA; alina.puig@usda.gov

**Keywords:** *Lasiodiplodia*, *Diaporthe*, *Phytophthora*, cacao diseases, cacao industry, Puerto Rico cacao

## Abstract

Cacao production is a rapidly expanding industry in Puerto Rico, with new farmers planting ~20,000 trees in the past few years. To determine the etiology and extent of diseases affecting cacao in Puerto Rico, a survey was performed at eight sites around the island. Pod rot and/or branch dieback were observed at all sites. Most organisms isolated from symptomatic pod and stem samples were identified as *Diaporthe* spp. (48%) and *Lasiodiplodia* spp. (25%) based on sequences of the internal transcribed spacer and large subunit regions. Within these genera, *Diaporthe tulliensis* and *Lasiodiplodia theobromae* were the most prevalent species and were used in inoculation studies to determine their relative virulence on pods and stems. *Phytophthora palmivora* served as a positive control due to its well-established pathogenicity in all tissues. On pods, *L. theobromae* and *P. palmivora* caused significantly larger lesions (6.1 and 5.9 cm, respectively) than *D. tulliensis* (2.7 cm) four days post-inoculation. All three species caused disease on stems, with no differences found among species. Although *P. palmivora* was thought to be the primary pathogen affecting cacao in Puerto Rico, this study identifies *L. theobromae* and *D. tulliensis* as the common pathogens on the island. This improved understanding will help scientists and farmers control disease by selecting fungicides effective against both oomycetes and fungi.

## 1. Introduction

The tropical tree, *Theobroma cacao*, is grown commercially for its seeds, which are used as the main ingredient in the global multibillion-dollar chocolate industry [1]. *T. cacao* originated in the Amazon rainforest, but the majority is now being produced outside its native range, with African farmers growing over 73% of the global crop [2]. The Americas account for only 17% of global production, but 80% of fine-flavor cacao beans are grown here [3]. Fine-flavor cacao beans have more desirable sensory and flavor profiles than bulk cacao beans, and they have a higher market price [4,5]. Ecuador exports 56% of global fine-flavor beans, followed by the Dominican Republic (19%) and Peru (12%) [5].

Oomycetes, fungi, and/or viruses cause diseases in all production areas, resulting in losses of up to 40% of the global harvest [6]. Some diseases, like black pod rot, caused by oomycetes in the *Phytophthora* genus, are present in all production areas worldwide, while others are geographically limited such as vascular streak dieback in Asia and cacao swollen shoot virus disease in Africa [7]. In the Americas, frosty pod rot (caused by *Moniliophthora roreri*) and witches’ broom (caused by *Moniliophthora perniciosa*) are responsible for significant yield losses. The distribution of these fungi within the Americas is currently limited, but they can be easily introduced with the movement of infected plant material or windblown spores [8,9,10].

Local cacao cultivation in Puerto Rico began in 1636, but adverse weather and a disease of unknown etiology prevented further development of the crop [11,12]. The United States Department of Agriculture (USDA) conducted cacao research in Puerto Rico throughout the 1900s. Promising clones were evaluated in a multisite trial, and nine were recommended to local farmers for planting [13]. In 2000, a large increase in commercial production occurred following an initiative to develop a cacao industry on the island. The industry on the island has rapidly expanded, with tens of thousands of trees being planted in the past years [14].

Despite the recent success of Puerto Rico’s cacao industry, few studies have been conducted to determine the diseases present on the island. All commercial farmers on the island are growing this crop for the first time, and due to the previous failure of the industry in the 1800s from unknown diseases, it is important to know what pathogens are present in the area. Until recently, black pod rot, caused by *Phytophthora palmivora*, was thought to be the primary disease affecting cacao on the island, and more information is needed on other pathogens that are present [15]. Several high-consequence pathogens, such as *M. perniciosa*, *M. roreri*, and *Ceratocystis cacaofunesta* are present in the Americas, including nearby Jamaica (*M. roreri*), St. Lucia (*M. perniciosa*), and Trinidad *(Ceratocystis cacaofunesta* and *M. perniciosa*) [16,17,18]. Symptomatology can be highly variable, making it important for identification to be made based on genetic sequences from isolated organisms. The objective of this study was to identify pathogens affecting *Theobroma cacao* in Puerto Rico by sampling at eight sites throughout the island, including five commercial farms, two research sites, and one forested area with naturalized cacao trees.

## 2. Results

### 2.1. Disease Prevalence and Pathogen Isolation

**Figure 1:**
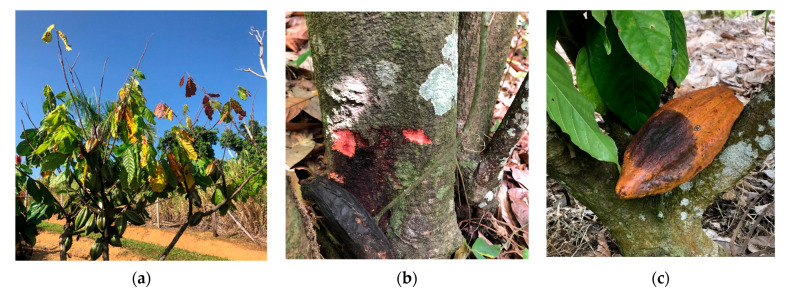
Diseases affecting cacao trees observed during this study. (**a**) Severe branch dieback on a tree growing in full sun; (**b**) bleeding canker with red stem tissue underneath bark; and (**c**) a pod with necrotic lesion.

Dieback, stem canker, and pods with necrotic lesions were observed on cacao trees at nearly all survey sites (Figure 1). The exception was the forested area with naturalized cacao trees, where no pods were present. Three of the sites had low to no shade. At these sites, severe dieback was observed on over 40% of trees. Most sites had relatively low incidences of pod rot with less than 10% of the trees having affected pods, except for the USDA germplasm collection, which had diseased pods on approximately 25% of the trees. Thirty-two pods and 45 stems/branches were sampled and processed for pathogen isolation. Fungal-like organisms were obtained from 24 out of 32 pods and 41 out of 45 stems sampled. Most of the pods yielded a single organism compared with only half of the stem samples.

**Figure 2:**
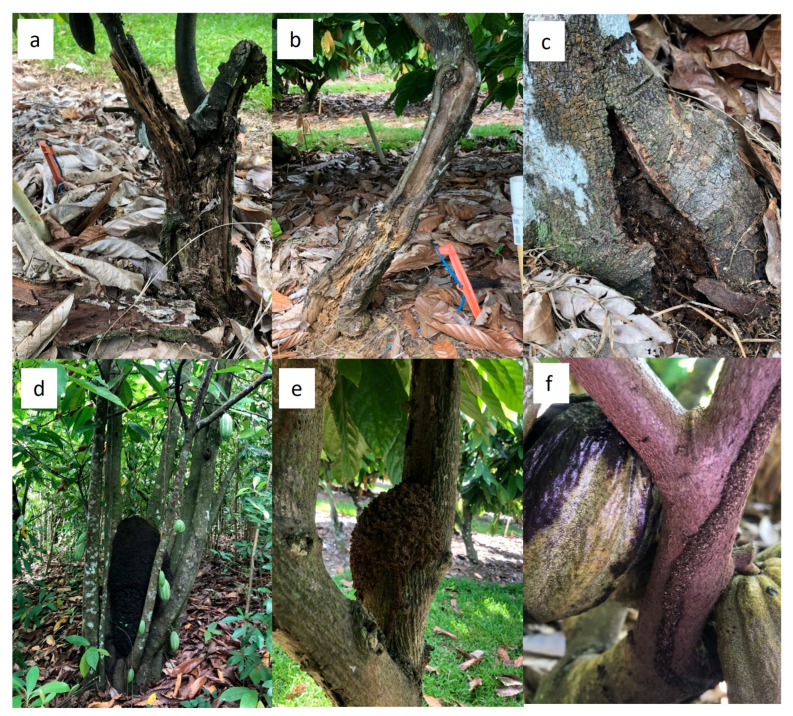
Termite damage on cacao trees in Puerto Rico: (**a**,**b**) living trees with visible termite galleries showing reduced stem volume and canopy; (**c**) termite colony visible through a crack at the stem base; (**d**,**e**) arboreal termite nests; and (**f**) termite mud tube on a tree branch.

Severe tree decline at multiple sites was accompanied by termite infestations. Many infested trees had lost over 50% of their stem tissue and branches, with little canopy remaining (Figure 2a,b). On other trees, termite colonies were visible through cracks in the bark at the stem base (Figure 2c). Arboreal termite nests and mud tubes were observed in several trees (Figure 2d–f).

A small number of trees with virus symptoms were observed during this study and found to be infected with cacao mild mosaic virus (CaMMV), as described in Puig et al. [19].

### 2.2. Molecular Identification

The most frequently isolated organisms were identified as *Diaporthe* spp. (47.9%) and *Lasiodiplodia* spp. (25.4%) based on genetic sequences. A subset of samples yielded *Fusarium* (11%), *Nigrospora* (11%), *Colletotrichum* (9%), and *Phytophthora palmivora* (7%); however, the first two were only found in stem samples (Table 1). *Phytophthora palmivora*, *Auricularia* sp., *Annulohypoxylon stygium*, *Cophinforma atrovirens*, *Curvularia* sp., *Exoserohilum rostratum*, *Daldinia eschscholtzii*, *Nectriaceae* sp., *Xylaria* sp., and *Neofusicoccum* sp. were obtained from diseased stem samples only once each. *Cophinforma atrovirens* was also obtained from a single diseased pod sample.

Within the *Lasiodiplodia* genus, *L. theobromae* was the predominant species recovered in this study, comprising 83.3% of all isolates. In addition, three isolates of *L. pseudotheobromae* and one of an undefined species were also recovered. Sequences from regions of the Internal Transcribed Spacer (ITS) and Large Subunit (LSU) of the latter, PR33, shared 100% identity with multiple species such as *L. parva* (MZ182360, JQ659278, etc.) and *L. citricola* (MN540684, MH183370, etc.).

Five of the thirty-four isolates of *Diaporthe* were identified as *D. pseudomangiferae* based on 100% identity with numerous entries in GenBank (MG576129), including one from Puerto Rico (KF616500). A single isolate was identified as *Diaporthe fraxini-angustifoliae* based on 100% identity with isolates from Panama (MF495396) and the USA (KU593528). Eight isolates were not identified to the species level, with most matching sequences having only genus-level identifications. Sequences from two representative isolates of these were deposited in GenBank as *Diaporthe* sp. 1 and *Diaporthe* sp. 2 (Table 2, Table S1).

### 2.3. Pathogenicity

**Figure 3:**
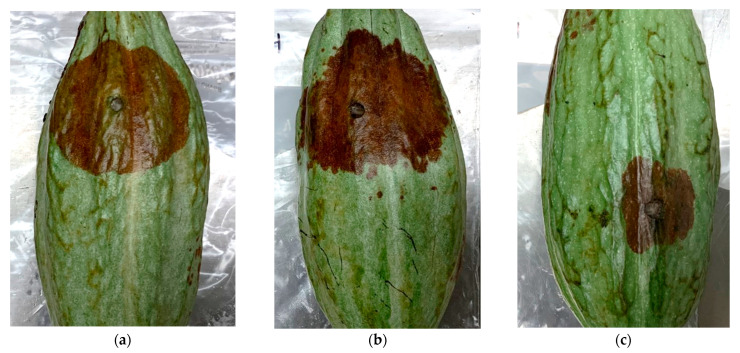
Necrotic lesions produced on cacao pods four days post-inoculation with (**a**) *Phytophthora palmivora*; (**b**) *Lasiodiplodia theobromae*; (**c**) *Diaporthe tulliensis*. Despite size differences, all three pathogens induced lesions of similar color and shape.

**Figure 4:**
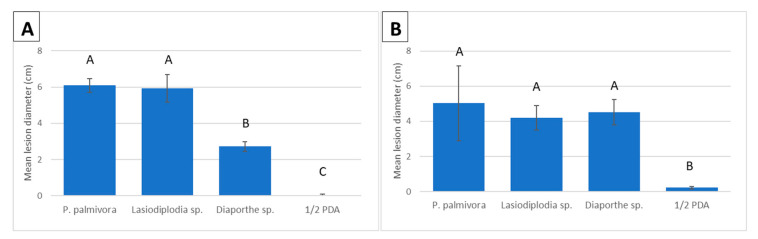
Average lesion diameter on pods (**A**) and stems (**B**) following inoculation with *Phytophthora palmivora*, *Lasiodiplodia theobromae*, *Diaporthe tulliensis*, and ½ PDA. The positive and negative controls are *P. palmivora* and ½ PDA, respectively. Species with the same letters above them are not significantly different (*p* ≤ 0.05), and bars represent standard errors.

Inoculation studies were performed with the predominant pathogens found, with *P. palmivora* serving as the positive control and uncolonized plugs of ½ PDA as the negative control. All pods inoculated with *P. palmivora*, *L. theobromae*, and *D. tulliensis* began developing firm black lesions within two to four days. Symptoms could not be distinguished based on the pathogen species used (Figure 3). However, *L. theobromae* and *P. palmivora* caused significantly larger lesions (6.1 and 5.9 cm, respectively) than *D. tulliensis* (2.7 cm) four days post-inoculation (*p* < 0.0011 and *p* < 0.0046) (Figure 4A).

All three species also caused disease on stems, with lesions ranging from 4.2 cm (±0.7) to 5.0 cm (±2.1). However, no differences in lesion sizes were found on stems following inoculation with the three species. The only significant differences found during stem inoculations were between the negative control and the three pathogens (*p* < 0.0278) (Figure 4B).

## 3. Discussion

This study represents the first island-wide study to determine the diseases affecting farmers in Puerto Rico’s emergent cacao industry. Several high-consequence pathogens, such as *Moniliophthora perniciosa*, *M. roreri*, and *Ceratocystis cacaofunesta* are present on nearby islands and could be accidentally introduced with the movement of infected material or hurricanes. Fortunately, none of these were detected on the island. Their absence gives the Puerto Rico cacao industry an advantage over other locations.

*Diaporthe tulliensis* and *Lasiodiplodia theobromae* were the most common pathogens found in this study, causing disease on both stems and pods. *Diaporthe* species were previously recently reported as pathogens on cacao pods [20,21], but their virulence relative to other pathogens was not known. This study shows that, on pods, *D. tulliensis* produces slightly smaller lesions than *L. theobromae* and *P. palmivora*. However, this is offset by their overall prevalence, with *D. tulliensis* present in 50% of diseased pods in this study. On stems, *L. theobromae* and *D. tulliensis* were shown to be of similar virulence as *P. palmivora*, which has been reported to kill over 10% of cacao trees annually [22,23]. *L. theobromae* is known to cause disease on both pods and stems of *T. cacao*, with substantial yield losses and tree death reported [24].

The *Diaporthe* genus includes species of *Diaporthe* as well as *Phomopsis*, which is its sexual state [25]. They are among of the most frequently encountered endophytes of cacao and other plants but are also common pathogens of plants and occasionally humans [26,27,28,29,30,31]. Villavicencio et al. [30] tested the pathogenicity of numerous cacao endophytes, and five out of nine *Diaporthe* spp. isolates were found to be pathogenic on fruit or leaves. Pathogenicity on woody tissue was not tested. This study is the first report of *D. tulliensis* as a stem pathogen on *T. cacao*.

A search of ‘cacao’ and ‘diaporthe’ in GenBank shows 103 deposited sequences from several species associated with the crop (NCBI, http://www.ncbi.nlm.nih.gov (accessed on 27 October 2023)). Most of these belong to *D. tulliensis* (67%) followed by *Diaporthe* sp. (17%) and then *D. pseudomangiferae* (9%). *D. tulliensis* is associated with diseased cacao in Malaysia (ON932315.1), Puerto Rico (OL412432), and Australia (NG_059130) [21,32]. Sequences of cacao-associated *D. phaseolorum* are available from Venezuela (KU377473) and Brazil (AY745987), *D. helianthi* from Brazil (AY746005), and *D. inconspicua* from Panama (MF495476). Sequences of *Diaporthe* sp. isolates with only genus-level identifications are available from cacao in China (KX999189) and soil from cacao production areas in Colombia (MN602872).

Despite only being recovered from two pods in this study, several species of *Colletotrichum* are known to cause disease in cacao fruits. *Colletotrichum siamense* and *C. tropicale* were recently reported as causing pod disease in Puerto Rico [33] and *C. gloeosporioides* in Cameroon, where its prevalence ranged between 18 and 39% [34]. However, in addition to being scarce, in this study, *Colletotrichum* spp. co-occurred in pods with *D. tulliensis* in both instances.

There is a substantial overlap between genera of pathogens and endophytes found in cacao. For example, *Lasiodiplodia*, *Diaporthe*, and *Colletotrichum* are among the most prevalent endophytes found in cacao tissue [31]. It is believed that these organisms may become pathogenic under certain conditions [35]. More information is needed on the effect of environmental conditions or abiotic stress on the ability of these organisms to cause disease.

Although the number of sites included in this study was sufficient to provide a general prevalence of disease and pathogen species, surveys are needed at different parts of the year to confirm these results. This study was conducted during the rainy season, which is expected to have favored the development of *Phytopthora* spp. diseases. Despite this, the overall prevalence of *P. palmivora* on pods was low. It is possible that this pathogen is relatively rare during the dry season. Additional studies are needed to confirm this.

The severity of termite damage observed in this study warrants additional research on this pest in Puerto Rico. Although historically, it was believed that trees attacked by termites were dying prior to the attack [36], numerous studies have shown that healthy living trees are also vulnerable to attack [37,38,39]. Several species of Coptotermes are known to target living trees [40], including *C. gestroi*, which is present in Puerto Rico [41]. Termites are a major constraint to cacao production in Africa, where their negative impacts on yield and tree survival have been extensively documented [42,43,44,45].

*Neofusicoccum parvum* was found to be a common cause of pod rot on cacao in Hawaii [46,47], but was not detected during this study. This may be due to the warmer climate of Puerto Rico compared with Hawaii, as *N. parvum* has a lower temperature optimum and range than *L. theobromae*, which was frequently detected in this study [47]. The related *Neofusicoccum mangiferae* was detected in a single sample. This tree was growing at the highest elevation production area on the island, which experienced substantially lower temperatures.

The role of fungi detected in only one or two samples is not clear. Some of these, such as *Annulohypoxylon stygium* (*Xylariales, Ascomycota*) are known wood decomposers [48], and are unlikely to have contributed to disease. *Xylaria* sp. are endophytic fungi of several plant species including cacao [31], as well as wood decomposers strongly associated with termites [49]. Nearly 70% of nests in South Africa contained this fungus, which is believed to be a termite symbiont [50]. Members of the *Curvularia, Cophinforma*, and *Nigrospora* genera have been reported as members of the microbial community of cocoa beans during the fermentation process [51]. However, *C. atrovirens* has been reported to cause dieback and canker on numerous woody plants such as cashew [52] and was recently found to be associated with dieback of *T. cacao* in Venezuela [53]. If these organisms are obtained from additional diseased material in the future, pathogenicity studies should be conducted to assess the relative risk they pose.

## 4. Materials and Methods

### 4.1. Pathogen Isolation

To determine what diseases are present on the island, eight sites were visited in August 2019: a USDA *T. cacao* germplasm collection in Mayaguez, a USDA field trial at Corozal Agricultural Experiment Station, a forested area with naturalized cacao trees, and five commercial farms (at high and low elevation). Diseased pods and stems were sampled and processed for pathogen isolation, and non-disease issues present at each site were recorded.

Samples of diseased pods were taken by excising 3 × 15 × 3 mm^3^ pieces from lesion margins containing healthy and symptomatic tissue. They were surface disinfested by immersing in 70% ethanol for 20 s, air dried on autoclaved paper towels, and plated on ½ strength potato dextrose agar (PDA) (Sigma Chemical Co., St. Louis, MO, USA; 19.5 g PDA, 7.5 g agar, and 1 L distilled water).

**Figure 5:**
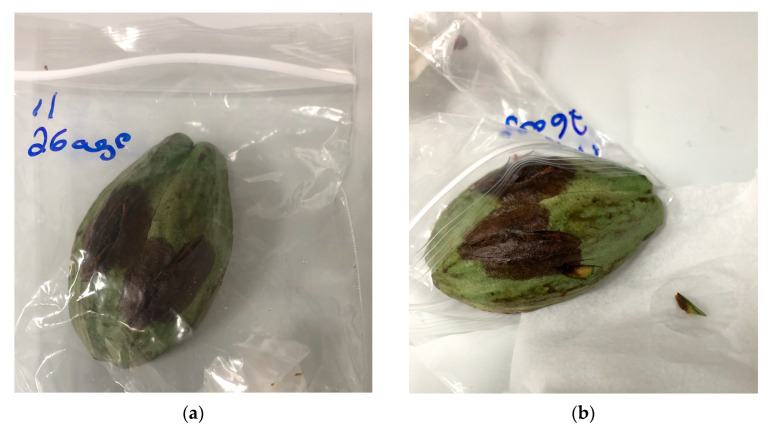
(**a**) Pathogens were isolated from diseased stems by surface-sterilizing and inserting tissue into a healthy cacao pod. (**b**) Newly emerging lesions were surface-disinfested and plated on ½ PDA.

Diseased stem samples were taken from the expanding edge of cankers and contained both healthy and symptomatic tissue. Due to the presence of numerous saprophytes and endophytes in the vascular tissue of *T. cacao* trees, stem samples underwent an additional step to improve the likelihood of recovering pathogens. Instead of plating tissue directly on media as described above, they were inserted into openings made in healthy 4-month-old cacao pods, placed in zip lock bags, and incubated at room temperature (28 ± 3 °C) as described in Puig et al. [47] (Figure 5a). After three days, tissue was sampled from the margins of newly emerging lesions, surface disinfested, and plated on ½ PDA media (Figure 5b) as described above.

After three to five days, emerging hyphae were subcultured onto new ½ PDA plates to obtain pure cultures, and then incubated at room temperature for one week prior to DNA extraction. Mycelia were harvested by scraping the surfaces of colonized plates and DNA was extracted using the Qiagen DNeasy Plant Mini Kit following the manufacturer’s protocol (Qiagen, Valencia, CA, USA).

### 4.2. Molecular Identification

Organisms were identified based on sequences from Internal Transcribed Spacer (ITS) and Large subunit (LSU) using primers ITS5 (5′ GGA AGT AAA AGT CGT AAC AAG G 3′) and ITS4 (5′ TCC TCC GCT TAT TGA TAT GC 3′) for the ITS region [54] and primers LR5 (5′ ATC CTG AGG GAA ACT TC 3′) and LR0R (5′ GTA CCC GCT GAA CTT AAG C 3′) for the LSU gene [55] (Table 3). Gene regions were amplified in 25 µL PCR reaction consisting of 1 μL of DNA template, 12.5 μL 2× Immomix Red (Bioline, Taunton, MA, USA), 1 μL each of 10 μm forward and reverse primer, and sterile nuclease-free water to 25 μL.

For ITS, amplification was achieved using the following thermocycler conditions: 95 °C for 10 min; then, 30 cycles of 95 °C for 30 s, 57 °C for 30 s, and 72 °C for 45 s followed by a final primer extension step of 72 °C for 5 min. LSU regions were amplified using the following touchdown program: 95 °C for 12 min and then 34 cycles of 95 °C for 70 s, 59 °C for 60 s (decreasing by 0.1 °C/cycle), and 72 °C for 80 s followed by a final primer extension step of 72 °C for 15 min. A Bio-Rad C1000 Touch thermal cycler (Bio-Rad Laboratories, Inc., Hercules, CA, USA) was used to run all PCRs. Amplified products were visualized on 1% (*w*/*v*) agarose gels containing 8 μL of Biotium GelRed (Biotium, Fremont, CA, USA).

PCR-amplified fragments were purified using the Qiagen PCR Purification Kit (Qiagen, Hilden, Germany) and then bi-directionally Sanger sequenced by Eurofins Genomics (Louisville, KY, USA). Forward and reverse sequences were aligned and edited using Geneious 11.1.2 (Biomatters Ltd., Auckland, New Zealand) and then analyzed in BLASTn to determine identity. Sequences from representative isolates recovered in this study were deposited in GenBank, and their accession numbers are listed in Table 2. Isolates matching sequences from multiple species at 100% identity, are identified by genus name only. Also, isolates only matching sequences with genus-level identifications are classified in the same manner. Prevalence of each organism was calculated as the number of samples it was isolated from dived by the total number of samples taken during this study. This was then converted to percentage by multiplying by 100.

### 4.3. Pathogenicity

The most prevalent species, *D. tulliensis* and *L. theobromae*, were used in inoculation studies to determine their relative virulence on pods and stems. Two isolates of each were tested alongside *Phytophthora palmivora* (isolate H33 from cacao in Hawaii [56]) which served as a positive control due to its well-established pathogenicity on all cacao tissues. Mycelial discs of uncolonized ½ PDA were used as the negative control.

Five-month-old open-pollinated pods of the clone CCN 51 were wounded with a 1 mm diameter probe, to a depth of 2 mm, and 6 mm discs of ½ PDA with actively growing mycelia of each isolate were placed mycelial-side down over the site, covered with a damp 1 × 1 cm^2^ filter paper square, and wrapped in parafilm, as described in Puig et al. [47]. They were placed in laboratory growth chambers at 25 °C in the dark. Four days post-inoculation, two diameters were measured for each lesion. These were averaged to calculate mean diameters (Table S1).

To determine pathogenicity on stems, three-month-old open-pollinated seedlings of the Amelonado variety (*n* = 19) were inoculated, as described above, and then placed in laboratory growth chambers at 25 °C on a cycle of 12 h light and 12 h darkness. Lesions were measured three weeks post-inoculation, by removing bark and measuring vertical and horizontal lesion diameter (Table S1).

To determine whether lesion sizes differed among genera, an analysis of variance (ANOVA) was conducted on average lesion diameters using PROC MIXED in SAS Ver. 9.4 (SAS Institute 2016, Cary, NC, USA). Means were separated using Tukey–Kramer Comparison lines for least squares means of the isolates. ANOVAs were conducted for pods and stems separately. To confirm that the resulting lesions on pods and stems were caused by the organism it had been inoculated with, isolations were made from a subset of lesions, as described above.

## 5. Conclusions

This research improves our understanding of the diseases and pests affecting cacao production in Puerto Rico and identifies *Diaporthe* and *Lasiodiplodia* as the most prevalent pathogen genera. No symptoms consistent with frosty pod rot, witches’ broom, or Ceratocystis wilt were observed in this study, providing additional evidence for their absence from the island.

Future work should focus on identifying sources of genetic resistance to the dieback pathogens found in this study. The severe dieback and canopy loss associated with termite colonization also warrants further study. Understanding the issues affecting cacao production in Puerto Rico will ensure the continued success of this emerging industry.

## Figures and Tables

**Table 1:** Genera recovered from diseased cacao material and the percentage of samples from which they were recovered. Genus determinations were made based on the best matches obtained with sequences of the Internal Transcribed Spacer (ITS) and Large Subunit (LSU) regions.

	Diaporthe	Lasiodiplodia	Colletotrichum	Phytophthora	Fusarium	Nigrospora
Pod (*n* = 26)	57.7% (15)	23.1% (6)	7.7% (2)	15.4% (4)	0	0
Stem (*n* = 45)	42.2% (19)	26.7% (12)	8.9% (4)	2.2% (1)	17.8% (8)	17.8% (8)

**Table 2:** Sequences of representative isolates of *Lasiodiplodia* spp. and *Diaporthe* spp. obtained in this study and deposited in the GenBank public database.

Species	Isolate	ITS	LSU
*Lasiodiplodia theobromae*	PR24	OR717218	OR717152
*Lasiodiplodia pseudotheobromae*	PR46	OR717219	OR717153
*Lasiodiplodia* sp.	PR33	OR717220	OR717154
*Diaporthe tulliensis*	PR14	OR717221	OR717155
*Diaporthe pseudomangiferae*	PR42B	OR717222	OR717156
*Diporthe* sp. 1	J1B	OR717223	OR717157
*Diporthe* sp. 2	PR32	OR717224	OR717158

**Table 3:** Primers used for the identification of fungal genera obtained in this study.

Locus	Primer Name	Primer Sequence (5′–3′)	Reference
LSU	LR5	ATCCTGAGGGAAACTTC	[55]
	LR0R	GTACCCGCTGAACTTAAGC	
ITS	ITS5	GGA AGT AAA AGT CGT AAC AAG G	[54]
	ITS4	TCC TCC GCT TAT TGA TAT GC	

## Data Availability

Sequence and inoculation data are available in Supplementary Tables S1 and S2.

## Supplementary Materials

The following supporting information can be downloaded at: https://www.mdpi.com/article/10.3390/plants12223855/s1, Table S1: Nucleotide sequences submitted to GenBank. Table S2: Pod and stem inoculation data.
